# *Callosphecodes*, a little-known bee (Hymenoptera, Halictidae, Sphecodes)

**DOI:** 10.3897/zookeys.127.1670

**Published:** 2011-09-08

**Authors:** Claus Rasmussen, Charles D. Michener

**Affiliations:** 1 Department of Biological Sciences, Aarhus University, Ny Munkegade 114, DK-8000 Aarhus C, Denmark; 2 Division of Entomology, Natural History Museum and Dept. of Ecology and Evolutionary Biology, 1501 Crestline Drive, University of Kansas, Lawrence, Kansas 66045, USA

**Keywords:** Apoidea, Anthophila, Halictinae, Halictini, Sphecodina, *Sphecodes*, New Britain, Bismarck Archipelago, Australia, taxonomy, cleptoparasite

## Abstract

*Callosphecodes* Friese, 1909, a synonym or perhaps subgenus of *Sphecodes* Latreille, 1804, is known on the basis of one female of *Sphecodes ralunensis* (Friese, 1909)from New Britain and one female and one male of a similar species, *Sphecodes manskii* (Rayment, 1935) from northeastern Australia. The male is here described for the first time and the females of the two species are compared for the first time. In spite of considerable collecting, only these three specimens have appeared in over a century. Descriptions and illustrations are provided.

## Introduction

Even in parts of the world where there has been little investigation of the bee fauna, taxa of bees so distinctive as to have received genus-group names a century or more ago have usually been collected several times so that multiple specimens are now known. *Callosphecodes* Friese, 1909, however, until now has been known from only two female specimens of different species from localities over 1500 km apart. A third specimen, a male, is herein reported for the first time. To judge by the lack of pollen manipulating and carrying structures in females, this is a cleptoparasitic group. Many cleptoparasites are uncommon, and it seems possible that *Callosphecodes* is a rare insect, not only in collections but also in the field.

We follow various earlier authors in considering *Callosphecodes* to be a synonym or possibly a subgenus of *Sphecodes* Latreille, 1804, which is the most common and widespread genus of cleptoparasitic Halictinae. This cleptoparasitic group was given subtribal status as the Sphecodina in the tribe Halictini (subfamily Halictinae) in the phylogenetic study by Pesenko (2000). Nonetheless, the two species that have been placed in *Callosphecodes* have a distinctive appearance different from that of the many other species of *Sphecodes*. Such other species are 4 to 15 mm in length, usually black with a partly or wholly red metasoma, but males in particular may be entirely black. A female at a host cell destroys the egg of the host and replaces it with her own. Further information on *Sphecodes* biology can be found in works by Ordway (1964), Eickwort and Eickwort (1972), Torchio (1975), Sick et al. (1994), and summarized by (Michener (2000, 2007).

## History

*Callosphecodes* was proposed as a subgenus of *Sphecodes* by Friese (1909) but in the same paper, in describing the included species, *Callosphecodes* was treated as a genus. The only species included at that time was *Callosphecodes ralunensis* Friese (1909), based on a single female presumably from Ralum, New Britain, in the Bismarck Archipelago (04°21'S, 152°17'E ). By error, Friese (1925) indicated that *Callosphecodes* had been described from Australia in 1912. It was separated from typical *Sphecodes* by its large size (but it is much smaller than the larger typical *Sphecodes*) and by the metallic blue black metasoma. Meyer (1920) repeated Friese’s description and because of the metallic coloration, suggested that *Callosphecodes* was close to the neotropical genus *Temnosoma* Smith (1853). The latter, however, is a very different cleptoparasite of the halictid tribe Augochlorini.

Subsequent views on the position of *Callosphecodes* have varied from a distinct genus (Friese 1925) to synonymy with *Sphecodes* (Michener 1944, 1978, 2000, 2007) or a subgenus of *Sphecodes* (Michener, 1965). These viewpoints were not based on additional information about the type species, for the type and only known specimen of *Sphecodes ralunensis* was not reexamined. After inquiring about the specimen from personnel of the Magyar Természettudományi Múzeum, Budapest, and the Museum für Naturkunde der Humboldt-Universität, Berlin, (Michener (2000, 2007) concluded that the specimen was probably lost. It has been found, however, in good condition in the Berlin museum and was borrowed for study by C.R.; all the labels were illustrated by Rasmussen and Ascher (2008, fig. 8).

A second specimen of *Callosphecodes* was described as *Mellitidia manskii* (Rayment 1935) on the basis of a single female collected in 1934 by Martin J. Manski at Cairns (16°55'S, 145°46'E ) in northern Queensland, Australia. It is not clear why it was placed by Rayment in a nomiine genus whose females, unlike cleptoparasites, have a strong scopa. Placement of this species in the genus *Sphecodes* was by Michener (1965), who saw the type, but association with *Callosphecodes* was not certain since the type of *Sphecodes ralunensis* was not then available. The holotype of *Sphecodes manskii* is in the Australian National Insect Collection, Canberra, and has been borrowed by C.R. for direct comparison with that of *Sphecodes ralunensis*. They are very similar, certainly both constituting the *Callosphecodes* group. Michener (1978) and Cardale (1993) included *Sphecodes manskii* not merely in *Sphecodes* but in the subgenus *Sphecodes* s. str.

Also, in the Australian National Insect Collection, was found a male, judged on the basis of similarity to the female and on geography, to be *Sphecodes manskii*. It was collected in 1980 by Josephine C. Cardale in Mount Webb National Park (15.045S, 145.07E), Queensland, Australia, about 100 km from Cairns, the type locality for *Sphecodes manskii*.

When reviewing the cleptoparasitic groups of Halictidae, Michener (1978) differentiated the genera such as *Eupetersia* Blüthgen, 1928, from *Sphecodes*; see also (Michener (2000, 2007). It is apparent that *Callosphecodes*, contrary to earlier suggestions (Michener, 1978), is not the same as any such genera but, as we have indicated above, does not differ appreciably from ordinary *Sphecodes*. The principle difference mentioned in the literature between such *Sphecodes* and *Callosphecodes* is the metallic blue, greenish or purplish black metasoma of the latter, independently mentioned by both Friese and Rayment in describing the two species. Yet, at least at present, the metallic tints of the specimens are extremely feeble, scarcely detectable, the metasoma being essentially black.

## Description

**Figure 1–5.**
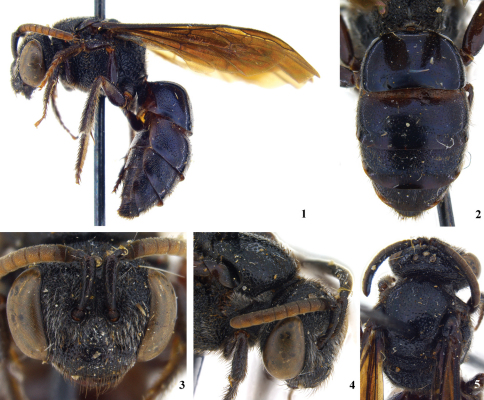
Holotype female of *Sphecodes ralunensis*: **1** lateral habitus **2** metasoma **3** facial aspect **4** dorsolateral aspect of head and pronotum **5** dorsal aspect of mesosoma and head.

**Figure 6–12.**
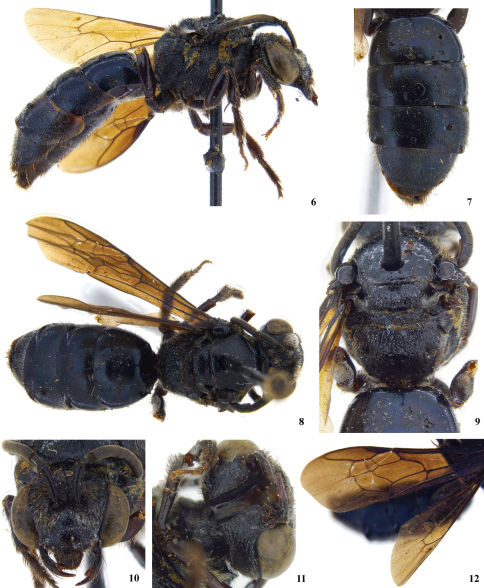
Holotype female of *Sphecodes manskii*: **6** lateral habitus **7** metasoma **8** dorsal habitus **9** mesosoma including propodeum **10** facial aspect **11** hypostomal carina with tooth at posterior end **12** forewing pattern.

**Figure 13–14.**
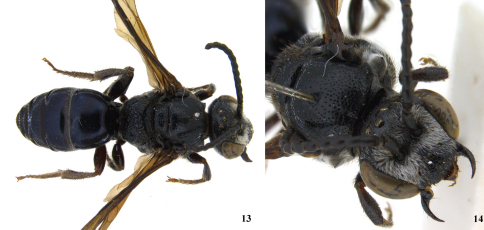
Male of *Sphecodes manskii*: **13** dorsal habitus; **14** dorsolateral aspect of head and pronotum.

The following descriptive comments, largely following the pattern of Michener’s (1978) account of *Sphecodes*, are based on the three known specimens of the *Callosphecodes* group, that is *Sphecodes ralunensis* and *manskii* (Figs 1, 6 and 13). The description of *Sphecodes* by Michener (1978) indicates the variation in many characters among the species of the genus. Notes below on the genus *Eupetersia* are inserted to counter the suggestion mentioned in (Michener (2000, 2007) that *Callosphecodes* might be a senior synonym of *Eupetersia*.

Both sexes: Black, metasomal terga with feeble bluish, purplish, or blue green metallic tints (Figs 2 and 7); wings strongly infuscated (fig. 12). Punctation of head and thorax coarse (fig. 4; moderately fine in *Eupetersia***);** punctures of mesoscutum, especially posteriorly, widely separated (by much more than puncture diameter) by shining surface (fig. 5). Head in facial view much wider than long, clypeus more than twice as wide as long (Figs 3 and 10). Eyes hairless. Hairs of antennal flagellum all very short. Preoccipital carina strong and distinct. Posterior end of hypostomal carina with tooth (fig. 11). Pronotum with horizontal surface of collar almost absent medially, forming lateral angle below which a vertical ridge extends downward; vertical ridge approaching or merging with a more laterally directed ridge that extends toward coxal base; another carina from lateral angle extends across posterior lobe of pronotum. Anterior extremity of mesoscutum convex. Scutellum gently biconvex because of feeble longitudinal median depression. Propodeum with dorsal area strongly areolate, about as long as scutellum, area broadly rounded posteriorly (fig. 9); posterior and lateral surfaces of propodeum with few short plumose hairs in addition to longer hairs. Wings with hairs rather long and dense throughout (as in *Eupetersia*); stigma moderate; marginal cell pointed at apex; free part of marginal cell beyond submarginal cells longer than part subtended by submarginal cells, which part extends well beyond apex of stigma. Second and third submarginal cells each receiving a recurrent vein (fig. 12). First metasomal tergum broader than long. Second tergum in lateral view with base somewhat depressed forming weak constriction between first and second terga. Posterior margins of terga 2 – 4 broadly depressed, hairless, impunctate.

Female: Mandible with large subapical tooth (fig. 10; unlike *Eupetersia*). Labrum with broad, flat apical process about two thirds as broad as long. Legs robust, hind femur about three times as long as broad; basitibial plate elevated; long hairs on outer side of hind tibia plumose; hind tibial spine finely serrate. Fifth metasomal tergum, unlike preceding terga, with apical margin fringed except middle part of margin which has smooth, hairless area in front of fringeless part of margin. Pygidial plate broader than in *Eupetersia*.

**Figure 15–16.**
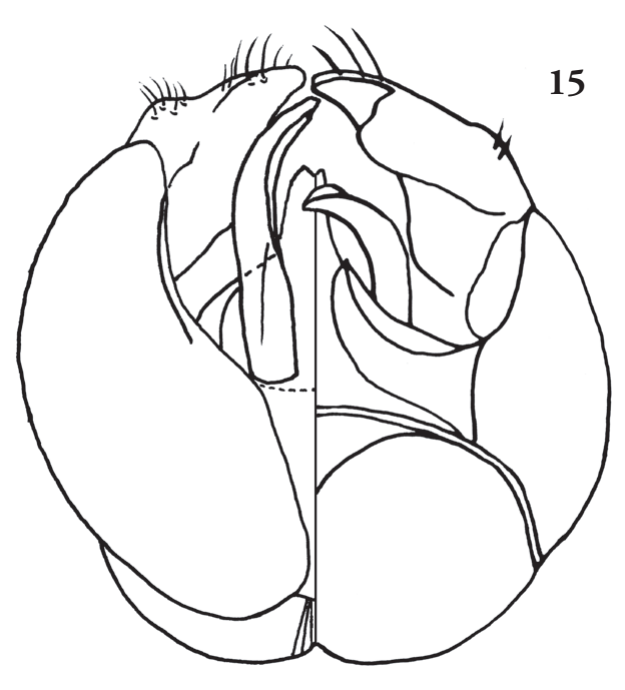
Male genitalia of *Sphecodes manskii*: **15** Dorsalventral view of genitalia; **16** 7th metasomal sternum.

Male: Antennae longer than those of female, flagellum thickened (fig. 13; unlike *Eupetersia*), somewhat crenulate, first flagellar segment broader than long, second longer than first, both first and second shorter than subsequent segments but not very short as in *Eupetersia*. Labrum not visible on specimen. Second hind tarsal segment longer than third, base broader than base of third. Gonocoxite finely striate, without margined depression as in *Eupetersia*. Gonocoxite with basal setose lobe (Figs 15 and 16).

Specific differences: The holotypes (both females) are very similar and we have no way of knowing whether the differences between them are specific differences or indicate variation within a species. The differences (observed by CR) are as follows: Lateral margin of propodeum (immediately below metanotum) in *Sphecodes ralunensis* largely areolate, in *Sphecodes manskii* widely strigulate and less areolate. Gena of *Sphecodes ralunensis* sparsely covered with plumose, light colored setae, in *Sphecodes manskii* densely covered with white setae. Flagellum in *Sphecodes ralunensis* ferruginous (fig. 3), in *Sphecodes manskii* dark brown (fig. 10). Measurements are as follows for the *Sphecodes ralunensis* holotype female: Total body length about 10 mm; forewing length (including tegula) 8.8 mm; head width 3.1 mm; head length (anterior margin of clypeus to summit of vertex) 2.5 mm; mesoscutum width 2.1; mesoscutum length 2.0 mm. The *Sphecodes manskii* holotype female: Total body length about 12 mm; forewing length (including tegula) 9.5 mm; head width 3.2 mm; head length (anterior margin of clypeus to summit of vertex) 2.5 mm; mesoscutum width 2.3; mesoscutum length 2.0 mm. Male *Sphecodes manskii*: Total body length about 11 mm; forewing length (including tegula) 8.1 mm; head width 2.9 mm; head length (anterior margin of clypeus to summit of vertex) 2.5 mm; mesoscutum width 2.3; mesoscutum length 2.2 mm.
